# Artificial Sweeteners and Cardiovascular Risk in Hungary: Beyond Traditional Risk Factors

**DOI:** 10.3390/jcm14134641

**Published:** 2025-06-30

**Authors:** Battamir Ulambayar, Attila Csaba Nagy

**Affiliations:** 1 Department of Health Informatics, Faculty of Health Sciences, University of Debrecen, 4032 Debrecen, Hungary; ulambayar.battamir@etk.unideb.hu; 2 Coordinating Centre for Epidemiology, University of Debrecen Clinical Centre, 4032 Debrecen, Hungary

**Keywords:** cardiovascular disease, risk factors, artificial sweetener, European Health Interview Survey, Hungary

## Abstract

**Background/Objectives**: Cardiovascular disease (CVD) remains a leading cause of mortality in Hungary, with rising obesity and diabetes rates exacerbating the burden. Artificial sweeteners, promoted as healthier alternatives to sugar, have been linked to CVD risk in other populations, but evidence in Hungary is limited. This study aims to investigate the association between artificial sweetener use and CVD prevalence in a Hungarian population, independent of traditional risk factors, using data from the 2019 European Health Interview Survey (EHIS). **Methods**: This cross-sectional study analyzed EHIS data (n = 5603), categorizing participants by self-reported CVD diagnoses (hypertension, coronary artery disease, myocardial infarction, or stroke). Variables included artificial sweetener use, age, sex, education, income, smoking, alcohol consumption, physical activity, diabetes, and body mass index (BMI). Chi-square tests and multivariate logistic regression, adjusted for sampling weights, were employed to assess associations. **Results**: Of 5603 participants, 38.7% had CVD and 16.4% used artificial sweeteners. Older age, female sex, lower education, long-term smoking cessation, obesity, diabetes, and physical inactivity increased CVD risk, while moderate alcohol consumption and sports were protective. Artificial sweetener use was significantly associated with higher CVD prevalence (OR: 1.21, 95% CI: 1.01–1.46, *p* = 0.01), independent of other risk factors. **Conclusions**: Artificial sweetener use is associated with increased CVD risk in Hungary, suggesting a need for further research and public health strategies to address this potential risk.

## 1. Introduction

Cardiovascular disease (CVD) remains a predominant global health issue, representing one of the leading causes of mortality and morbidity across various populations [1,2], with a particularly high burden among older adults and those with multiple risk factors [3]. While CVD mortality has declined in Western Europe [4], Eastern European countries—including Hungary—continue to report higher rates, driven by smoking, hypertension, and unhealthy diets [5]. In Hungary, despite improvements in healthcare, CVD remains a major concern due to rising obesity, diabetes, and genetic conditions like familial hypercholesterolemias [6,7,8,9].

Artificial sweeteners, such as aspartame and sucralose, are widely consumed to reduce caloric intake and manage conditions like diabetes and obesity. The discussions surrounding artificial sweeteners and their potential links to CVD have garnered increasing attention in recent years. A recent study utilizing data from the UK Biobank suggested a significant association between artificial sweetener intake and the incidence of CVD and mortality [10]. Moreover, Witkowski et al. have emphasized the potential adverse effects of specific artificial sweeteners, such as erythritol, on CVD risk, suggesting that even widely recognized low-calorie alternatives may not be as benign as previously thought [11]. The effects of artificial sweeteners on cardiovascular health may also derive from their impact on metabolic processes [12]. Evidence from various cohort studies indicates that long-term consumption of artificially sweetened beverages correlates with increased weight gain and higher body mass index (BMI), both of which are recognized risk factors for CVD [13,14].

While the association between artificial sweeteners and CVD has been explored in various populations, evidence remains limited in specific cultural and dietary contexts such as Hungary. Hungary is characterized by distinct dietary habits, including a high intake of processed foods, meat, and added fats [15], alongside increasing consumption of “sugar-free” or “diet” products marketed as healthier alternatives all around the world [16]. At the same time, the country faces one of the highest CVD mortality rates in Europe and lags behind European averages in achieving target levels for LDL cholesterol and waist circumference in very-high-risk patients [17]. These factors, combined with unique regional dietary behaviors, underscore the importance of investigating whether artificial sweetener use contributes to cardiovascular risk independently of established risk factors. Understanding this relationship within the Hungarian context is essential for developing culturally appropriate dietary guidelines and informing public health strategies aimed at reducing the national CVD burden. This study aims to address this gap by analyzing data from the European Health Interview Survey (EHIS) to investigate the impact of artificial sweetener use on CVD prevalence in a Hungarian population.

## 2. Materials and Methods

### 2.1. Study Design and Data

This research employed a cross-sectional design, utilizing data collected in Hungary in 2019 as part of the EHIS. The data were collected under Eurostat’s oversight with a standardized questionnaire, and the sample (n = 5603) was adequately representative of Hungary’s general population [18]. Inclusion criteria comprised all participants aged 18 years or older from the 2019 Hungarian EHIS dataset with complete information on cardiovascular disease diagnosis and key covariates, including sweetener use, age, sex, BMI, smoking status, physical activity, and alcohol consumption. Participants with missing or incomplete data on any of these variables were excluded from the final analysis.

### 2.2. Variables

The study participants were divided into two groups: those with CVD and those without, based on self-reported diagnoses of hypertension, coronary artery disease, acute myocardial infarction, or stroke within the past 12 months. The self-reported diagnoses were validated by a follow-up question asking whether the diagnosis was confirmed by a doctor. The EHIS data included well-established CVD risk factors, such as age, sex, education, income level, smoking, alcohol use, obesity, physical inactivity, and salt intake, alongside artificial sweetener use, which is the primary focus of this research. Age was grouped into three categories: 34 years and younger, 35–64 years, and 65 years and older. Education level was classified as primary, secondary, or high (tertiary) education. Household income level was divided into five categories: lowest, lower than average, average, higher than average, and highest, based on net household income per capita, reflecting socioeconomic status.

Smoking status was categorized as active smokers or cessation within one year, cessation for more than one year, or never smoked in order to reflect long-term impact. Alcohol consumption was classified as heavy drinker (reference group), moderate drinker, or never/rare drinker based on self-reported alcohol consumption. Physical activity was assessed by the number of days per week participants engaged in walking, cycling, or sports for at least 10 min, each categorized as none (do not walk/cycle/perform sports), 1–3 days, or 4–7 days. Sweetener use for hot drinks (coffee, tea) was divided into natural (sugar, honey) or none and artificial sweetener use. Salt use was categorized as never/low or moderate/high. Body mass index (BMI) was classified as normal (≤24.9 kg/m^2^), overweight (25–29.9 kg/m^2^), or obese (≥30 kg/m^2^) [19].

### 2.3. Statistical Analysis

Data were analyzed using Chi-square tests to assess differences between participants with and without CVD, with *p*-values indicating statistical significance (*p* < 0.05). Multivariate logistic regression investigated associations between CVD and demographic, socioeconomic, and lifestyle factors. The model included the above-mentioned CVD risk factors in addition to artificial sweetener consumption based on the significant associations detected by the Chi-square test. Sampling weights were applied during the analysis to ensure that the results more accurately represented the target population’s characteristics, accounting for the study’s complex sampling design. Odds ratios (ORs) with 95% confidence intervals (CIs) were calculated to estimate the strength of associations, with *p*-values < 0.05 considered significant. STATA IC version 18.0 was used to conduct the statistical analyses used in this study [20].

## 3. Results

The study population comprised 5603 individuals, of whom 61.3% (n = 3436) had no cardiovascular disease (CVD) and 38.7% (n = 2167) had CVD. Among CVD-related conditions, hypertension was the most prevalent, affecting 30.61% (n = 1715) of the population, followed by coronary artery disease (CAD) at 4.30% (n = 241), acute myocardial infarction (AMI) at 2.68% (n = 150), and stroke at 2.32% (n = 130). The gender distribution was 45.9% males (n = 2572) and 54.1% females (n = 3031). Age distribution showed 22.8% (n = 1276) were aged 34 years and younger, 48.2% (n = 2699) were aged 35–64 years, and 28.7% (n = 1608) were 65 years and older. Regarding the use of artificial sweeteners, the primary focus of this study, 16.4% (n = 917) of the total survey participants reported regular consumption in coffee and tea.

As shown in Table 1, several demographic, socioeconomic, and lifestyle factors were significantly associated with CVD prevalence. Older age showed a strong association (*p* < 0.001), with CVD rates rising from 4.6% in those aged ≤34 to 72.9% in those ≥65. Females had a higher prevalence than males (41.0% vs. 35.0%, *p* < 0.001). Lower education and income levels were also linked to increased CVD risk, with prevalence reaching 46.5% among those with primary education and 42.8% in the lowest income group (*p* < 0.001 for both). Among lifestyle factors, obesity was strongly associated with CVD (58.1% in obese vs. 22.9% in normal BMI, *p* < 0.001), and physical inactivity correlated with higher prevalence across walking, cycling, and sports variables (all *p* < 0.001). Former smokers had the highest CVD rates (52.9%) compared to current/recent smokers (29.8%, *p* < 0.001), possibly reflecting cumulative exposure. Moderate alcohol use was linked to lower CVD prevalence (33.1%) versus heavy (39.5%) or rare/never drinkers (46.7%, *p* < 0.001). Notably, artificial sweetener users had significantly higher CVD prevalence (53.9%) than those using natural sweeteners or none (35.7%, *p* < 0.001), while salt use was not significantly associated with CVD (*p* = 0.424). For cardiovascular comorbidity, 78.2% of individuals with diabetes mellitus had CVD compared to 34.5% among those without diabetes (*p* < 0.001).

Table 2 presents the results of the adjusted logistic regression model, and it shows several factors were significantly associated with the CVD. Compared to males, females had higher odds of the outcome (OR = 1.32; 95% CI: 1.13–1.53; *p* = 0.001). Age showed a strong, graded association, with individuals aged 35–64 having over eight times the odds (OR = 8.27; 95% CI: 6.17–11.1; *p* < 0.001), and those aged 65 and older having nearly 36 times the odds (OR = 35.7; 95% CI: 26.2–48.6; *p* < 0.001) compared to those aged 34 or younger. Higher educational attainment was inversely associated with the outcome: individuals with secondary (OR = 0.66; 95% CI: 0.56–0.78; *p* < 0.001) and high-level education (OR = 0.58; 95% CI: 0.47–0.72; *p* < 0.001) had significantly lower odds than those with primary education. Smoking cessation for more than one year was associated with increased odds compared to daily smoking (OR = 1.28; 95% CI: 1.04–1.57; *p* = 0.018), whereas never smoking was not significantly associated. Among alcohol use categories, moderate drinkers had lower odds than heavy drinkers (OR = 0.84; 95% CI: 0.70–0.98; *p* = 0.044). BMI showed a significant relationship: overweight individuals had 1.67 times the odds (95% CI: 1.42–1.97; *p* < 0.001), and obese individuals had three times the odds (OR = 3.01; 95% CI: 2.52–3.60; *p* < 0.001) compared to those with normal weight. Physical activity in the form of sports was protective: performing sports 1–3 days per week was associated with 35% lower odds (OR = 0.65; 95% CI: 0.54–0.79; *p* < 0.001), and 4–7 days per week with 17% lower odds (OR = 0.83; 95% CI: 0.67–0.97; *p* = 0.021) compared to not performing sports at all. Individuals with diabetes mellitus had over three times the odds of CVD compared to non-diabetics (OR = 3.18; 95% CI: 2.47–4.08; *p* < 0.001). Lastly, using artificial sweeteners for tea or coffee was associated with higher odds of the outcome (OR = 1.21; 95% CI: 1.02–1.46; *p* = 0.01).

## 4. Discussion

In this study, we aimed to investigate the association between artificial sweetener use and CVD in the Hungarian population. The results of the study showed that artificial sweetener use increases CVD risk in addition to traditional cardiovascular risk factors.

Non-modifiable risk factors for CVDs are inherent characteristics that cannot be changed or controlled through lifestyle or medical interventions [21]. The most widely recognized non-modifiable risk factors include age, sex, family history of CVD, and genetic predisposition. Various studies confirm that the incidence of CVD continues to rise with advancing age, with heart disease becoming more prevalent in older populations [22]. Gender is another notable non-modifiable risk factor affecting CVD risk. Statistically, men have a higher incidence of CVD compared to women at younger ages [23]; however, post-menopause, the risk for women increases significantly, indicating that hormonal changes and socioeconomic status may influence cardiovascular health [24]. Therefore, gender differences in the prevalence of CVD tend to vary from country to country depending on the demographic structure and socioeconomic situation, and our study found that women are at higher risk, which is likely a demographic feature of Hungary, where the number of elderly people is increasing.

Recent studies consistently demonstrate an inverse relationship between socioeconomic status and CVD risk [25]. This association is particularly strong in high-income countries and among younger age groups [26]. Lower educational attainment significantly correlates with higher incident CVD globally, asserting that education acts as a robust predictor of CVD risk across multiple settings [27]. Lower socioeconomic status and limited education often lead to poorer access to healthcare, unhealthy lifestyle choices, higher chronic stress, and exposure to adverse neighborhood conditions, all of which contribute to increased CVD risk [28], consistent with our findings.

The modifiable risk factors of CVDs are of paramount importance in both prevention and management strategies. Our findings revealed significant associations between CVD and lifestyle-related risk factors, including obesity, diabetes, physical inactivity, alcohol use, and smoking. Obesity and diabetes contributes to cardiovascular risk both directly and indirectly through related conditions like hypertension and dyslipidemia, and weight reduction through lifestyle interventions remains a key prevention strategy [29,30]. Consistent physical activity enhances metabolic health and supports cardiovascular protection through improved fitness, lipid profiles, and weight control [31,32], as shown in our results, specially more intense physical activity. Smoking is a well-established risk factor for CVD [33] and smoking cessation programs have demonstrated effectiveness not only in reducing smoking prevalence but also in decreasing cardiovascular-related morbidity and mortality rates [34]. However, our results showed that individuals who quit smoking for over a year had 30% higher odds compared to daily smokers, while never-smokers showed no significant difference. The elevated CVD risk among long-term former smokers compared to current smokers may reflect the cumulative effects of prolonged tobacco exposure prior to cessation. Many individuals may quit smoking only after developing health complications, including early signs of cardiovascular disease, which could lead to a higher observed risk in this group despite their current non-smoking status. The lack of difference for never-smokers may reflect confounding factors or self-reported bias in smoking status. Alcohol consumption is a complex and multifaceted risk factor for CVD. The relationship between alcohol intake and CVD is often depicted through suggestive patterns, with certain levels of alcohol intake being associated with both protective and detrimental effects, particularly influenced by the quantity consumed and individual factors such as sex, age, and genetic predispositions [35]. Our findings align with previous studies suggesting a J-shaped relationship between alcohol consumption and CVD, where moderate intake may offer some protection compared to heavy drinking or abstinence [35,36,37].

Our findings indicate that artificial sweetener use in tea or coffee is associated with an increased risk of CVD, independent of the aforementioned modifiable and non-modifiable risk factors. The association between artificial sweetener use and CVD has become a significant area of research, particularly as the prevalence of artificial sweetener consumption has increased in recent years [38]. This trend is partly driven by widespread marketing efforts from food and beverage manufacturers, who promote “no sugar” or “sugar-free” products as calorie-free [39] and beneficial for weight management [40]. Such claims have led many consumers to perceive artificial sweeteners as a healthier alternative to sugar, especially in the context of obesity and diabetes prevention [41]. However, emerging evidence has raised concerns about potential adverse health effects associated with long-term consumption of these sugar substitutes, prompting further investigation into their safety and metabolic impact [42].

The relationship between artificial sweetener and CVD appears to be complex and multifaceted, involving metabolic pathways, dietary patterns, and potential physiological effects. Research indicates that individuals who regularly consume artificially sweetened beverages exhibit higher rates of obesity and metabolic syndrome [12,38]—conditions that are well-established risk factors for CVD. Artificial sweeteners like sucralose and aspartame have been shown to interact with sweet taste receptors in the gastrointestinal tract. This interaction could influence insulin secretion and glucose metabolism, potentially leading to insulin resistance over time [43,44]. Furthermore, the habitual intake of these sweeteners has raised concerns over their role in appetite regulation, as some studies suggest they may generate a disconnect between sweetness perception and caloric intake, thereby promoting overeating and weight gain [45]. Studies have also shown that the consumption of artificial sweeteners such as sucralose and aspartame can lead to changes in the microbial population, shifting toward dysbiosis—a state characterized by an imbalance in gut microbiota that can contribute to metabolic disorders, including obesity and type 2 diabetes [46,47].

However, our study results indicate that the effect of artificial sweeteners on CVD risk is independent of obesity and diabetes, suggesting that these metabolic conditions alone do not fully account for this association. Inflammation is another critical pathway identified in the literature regarding the association between artificial sweeteners and CVD risk. For instance, the consumption of artificially sweetened beverages has been linked to elevated markers of inflammation, which are significant contributors to CVD pathophysiology [48]. Emerging studies also point to direct cardiovascular effects of artificial sweeteners, such as acute cardiac toxicity or changes in cardiac performance. While the exact mechanisms remain to be fully explained, some researchers hypothesize that certain artificial sweeteners may have inherent toxic properties that could lead to cardiac dysfunction independent of traditional risk factors such as obesity or metabolic syndrome [49]. Another critical proposed mechanism is the induction of oxidative stress. Research indicates that artificial sweeteners can instigate an oxidative environment, increasing the production of reactive oxygen species in endothelial cells [50]. This oxidative stress can compromise endothelial function, leading to vascular reactivity impairments over time. We summarized the proposed mechanisms linking CVD and artificial sweetener use from the literature in the Supplementary Materials (Table S1). 

This study has several limitations. The data are based on self-reported responses from participants, which may be biased. A key limitation of our study is that the EHIS dataset does not differentiate between types of artificial sweeteners, and there is no information on the frequency and quantity of artificial sweetener consumption; it only captures general use in hot beverages. Future studies should aim to capture more detailed data on specific sweetener types, dosage, and duration of use to better characterize the exposure–outcome relationship and inform dietary guidelines. Given the cross-sectional nature of the EHIS dataset, this study cannot establish temporal or causal relationships between artificial sweetener use and CVD. While our findings highlight an association, future prospective cohort or case–control studies are needed to better assess causality. The primary strength of this study lies in its use of a weighted logistic regression model to assess the impact of artificial sweeteners on cardiovascular disease risk based on robust data collected by the Hungarian Central Statistical Office using a Eurostat-validated questionnaire and a representative sample of the Hungarian population.

## 5. Conclusions

This study highlights a significant association between artificial sweetener use and CVD prevalence in the Hungarian population, independent of well-established risk factors such as older age, lower education, smoking, heavy alcohol consumption, physical inactivity, obesity, and diabetes. While these findings suggest a potential link between artificial sweeteners and CVD, further longitudinal studies are needed to establish causality and explore underlying mechanisms, such as metabolic or behavioral factors. Public health interventions should focus on promoting awareness of potential risks associated with artificial sweetener consumption, encouraging balanced diets, and supporting lifestyle modifications like increased physical activity and weight management to reduce CVD risk.

## Figures and Tables

**Table 1:** Distribution of CVD prevalence by sociodemographic and lifestyle characteristics.

Variable	Category	CVD		*p* Value
		Without CVD	With CVD	
**Gender**	Male	1647 (64.0)	925 (35.0)	**<0.001**
	Female	1789 (59.0)	1242 (41.0)	
**Age group**	34 and younger	1217 (95.4)	59 (4.6)	**<0.001**
	35–64 years old	1777 (65.8)	922 (34.8)	
	65 and older	422 (27.1)	1186 (72.9)	
**Education**	Primary	1342 (53.5)	1165 (46.5)	**<0.001**
	Secondary	1216 (65.9)	628 (34.1)	
	High	878 (70.1)	374 (29.9)	
**Household income level**	Lowest	660 (57.2)	493 (42.8)	**<0.001**
	Lower than average	627 (53.4)	546 (46.6)	
	Average	682 (59.9)	457 (40.1)	
	Higher than average	803 (63.3)	466 (37.7)	
	Highest	664 (76.4)	205 (23.6)	
**Smoking status**	Active or cessation within a year	1086 (70.2)	462 (29.8)	**<0.001**
	Cessation for more than a year	463 (47.1)	520 (52.9)	
	Never smoked	1842 (61.3)	1163 (38.7)	
**Alcohol consumption**	Heavy drinker (Reference)	829 (60.5)	541 (39.5)	**<0.001**
	Moderate drinker	1651 (66.9)	818 (33.1)	
	Never of rare drinker	905 (53.3)	792 (46.7)	
**Number of days walked for at least 10 min a week**	Do not walk	366 (49.4)	375 (50.6)	**<0.001**
	1–3 days	502 (56.1)	393 (43.9)	
	4–7 days	2501 (65.5)	1375 (35.5)	
**Number of days cycled for at least 10 min a week**	Do not cycle	2068 (58.9)	1445 (41.1)	**<0.001**
	1–3 days	638 (68.6)	292 (31.4)	
	4–7 days	650 (62.2)	395 (37.8)	
**Number of days performing sports for at least 10 min a week**	Do not perform sports	1758 (52.7)	1580 (47.3)	**<0.001**
	1–3 days	1070 (77.3)	315 (22.7)	
	4–7 days	546 (69.1)	244 (30.9)	
**Servings of fruits a day**	1 serving	721 (55.0)	590 (45.0)	0.563
	2 servings	668 (52.9)	595 (47.1)	
	3 or more servings	309 (53.9)	264 (46.1)	
**Servings of vegetables a day**	1 serving	741 (57.6)	546 (42.4)	0.152
	2 servings	468 (56.5)	360 (43.8)	
	3 or more servings	211 (56.8)	161 (43.2)	
**Red meat use**	More than 4 times a week	444 (64.3)	246 (35.7)	0.177
	1–3 times a week	2019 (60.6)	1315 (39.4)	
	Less than one a week	928 (61.2)	588 (38.2)	
**White meat use**	More than 4 times a week	745 (58.0)	538 (42.0)	0.231
	1–3 times a week	2391 (59.8)	1607 (40.2)	
	Less than one a week	160 (57.3)	119 (42.7)	
**Milk products use**	More than 4 times a week	2166 (61.3)	1365 (38.7)	0.589
	1–3 times a week	895 (61.8)	553 (38.2)	
	Less than one a week	345 (59.4)	236 (40.6)	
**Sweetener for hot drinks (coffee, tea)**	Natural (sugar, honey) or not	3013 (64.3)	1673 (35.7)	**<0.001**
	Artificial sweetener	423 (46.1)	494 (53.9)	
**Salt use**	Never or low salt use	2389 (61.5)	1491 (38.5)	0.424
	Moderate or high salt use	1008 (60.4)	660 (39.6)	
**BMI**	Normal (≤24.9)	1699 (77.1)	506 (22.9)	**<0.001**
	Overweight (25–29.9)	1105 (56.9)	834 (43.1)	
	Obese (≥30)	584 (41.9)	809 (58.1)	
**Diabetes mellitus**	No	3265 (65.5)	1723 (34.5)	**<0.001**
	Yes	119 (21.8)	426 (78.2)	

Bold values indicate statistical significance (*p* < 0.05) based on Chi-square test.

**Table 2:** Multivariable logistic regression model.

Variable	Category/Level	OR	95% CI	*p*-Value
**Gender**	Male (Reference)			
	Female	1.32	1.13–1.53	**0.001**
**Age group**	34 and younger (Reference)			
	35–64 years old	8.27	6.17–11.1	**<0.001**
	65 and older	35.7	26.2–48.6	**<0.001**
**Education**	Primary (Reference)			
	Secondary	0.66	0.56–0.78	**<0.001**
	High	0.58	0.47–0.72	**<0.001**
**Income levels**	Lower than average (Reference)			
	Average	0.85	0.70–1.03	0.104
	Higher than average	0.98	0.83–1.16	0.874
**Smoking status**	Every day (Reference)			
	Cessation for more than a year	1.28	1.04–1.57	**0.018**
	Never smoked	1.07	0.90–1.27	0.407
**Alcohol use**	Heavy drinker (Reference)			
	Moderate drinker	0.84	0.70–0.98	**0.044**
	Never of rare drinker	0.95	0.78–1.16	0.664
**BMI**	Normal (Reference)			
	Overweight	1.67	1.42–1.97	**<0.001**
	Obese	3.01	2.52–3.60	**<0.001**
**Number of days walked for at least 10 min a week**	Do not walk (Reference)			
	1–3 days	0.96	0.75–1.24	0.805
	4–7 days	0.94	0.77–1.15	0.578
**Number of days cycled for at least 10 min a week**	Do not cycle (Reference)			
	1–3 days	1.01	0.83–1.22	0.916
	4–7 days	0.98	0.82–1.17	0.826
**Number of days performing sports for at least 10 min a week**	Do not perform sports (Reference)			
	1–3 days	0.65	0.54–0.79	**<0.001**
	4–7 days	0.83	0.67–0.97	**0.021**
**Diabetes mellitus**	No (Reference)			
	Yes	3.18	2.47–4.08	**<0.001**
**Sweetener use for tea or coffee**	Natural (Reference)			
	Artificial	1.21	1.02–1.46	**0.01**

Bold values indicate statistical significance (*p* < 0.05). Odds ratios are adjusted for variables in the model.

## Data Availability

The data analyzed in this study are subject to the following licenses/restrictions: The data presented in this study are available upon request from Hungarian Central Statistical Office who performed and supervised the data collection. Requests to access these datasets should be directed to Hungarian Central Statistical Office, www.ksh.hu/?lang=en (accessed on 5 May 2025).

## Supplementary Materials

The following supporting information can be downloaded at: https://www.mdpi.com/article/10.3390/jcm14134641/s1, Table S1: Summary of proposed mechanisms linking CVD and artificial sweetener use from the literature;
