# Supplementary material for: Artificial Sweeteners and Cardiovascular Risk in Hungary: Beyond Traditional Risk Factors
Source: J Clin Med. 2025 Jun 30;14(13):4641. doi: 10.3390/jcm14134641 (PMC12251530; doi:10.3390/jcm14134641)
Supplement: Supplementary file 1 [file jcm-14-04641-s001.zip › jcm-3713634-supplementary.pdf]

Supplementary Table S1. Summary of proposed mechanisms linking CVD and artificial sweetener use from the literature

Proposed Mechanism	Description	References
Altered glucose metabolism	Artificial sweeteners may affect insulin response and glucose tolerance	De Koning et al., 2011; Moriconi et al., 2020
Gut microbiota disruption	Changes in microbial composition may contribute to metabolic syndrome	Gopalakrishnan et al., 2024; Laforest-Lapointe et al.,2020
Inflammation	Intake linked to increased inflammatory markers associated with CVD risk	De Koning et al., 2012
Oxidative stress	Sweeteners may increase reactive oxygen species and impair endothelial function	Ibragić and Fehratović, 2021
Appetite regulation	Disruption in calorie-sweetness balance may promote overeating	Hernández et al., 2018
Direct cardiac effects	Some compounds may exert toxic or performance-altering effects on the heart	Saputra et al., 2020
